# In Vitro Antioxidant, Antithrombotic and Anti-Inflammatory Activities of Bioactive Metabolites Extracted from Kiwi and Its By-Products

**DOI:** 10.3390/metabo15060400

**Published:** 2025-06-13

**Authors:** Anastasia Maria Moysidou, Konstantina Cheimpeloglou, Spyridoula Ioanna Koutra, Vasileios Manousakis, Anna Ofrydopoulou, Katie Shiels, Sushanta Kumar Saha, Alexandros Tsoupras

**Affiliations:** 1 Hephaestus, Laboratory, School of Chemistry, Faculty of Sciences, Democritus University of Thrace, St. Lukas, 65404 Kavala, Greece; anastasiamoysidou02@gmail.com (A.M.M.); kocheib@chem.duth.gr (K.C.); spkoutr@chem.duth.gr (S.I.K.); vamanvu@chem.duth.gr (V.M.); anofrid@chem.duth.gr (A.O.); 2 Centre for Applied Bioscience Research, Technological University of the Shannon: Midlands Midwest, Moylish Park, V94 E8YF Limerick, Ireland; katie.shiels@tus.ie (K.S.); sushanta.saha@tus.ie (S.K.S.)

**Keywords:** anti-inflammatory, antioxidants, bio-waste, bioactive ingredients, by-products, cosmetics, functional foods, kiwi, phenolic compounds, platelet

## Abstract

**Background/Objectives:** Growing interest in natural, health-promoting ingredients for functional foods, nutraceuticals, and cosmetics has increased the demand for bioactive compounds from kiwi (Actinidia deliciosa). This study aimed to assess the antioxidant, anti-inflammatory, and antithrombotic properties of amphiphilic bioactives extracted from kiwi fruit and its by-products, including peel, seeds, and pulp. **Methods:** Bioactive compounds were extracted and analyzed using liquid chromatography–mass spectrometry (LC–MS) and attenuated total reflectance–Fourier transform infrared (ATR–FTIR) spectroscopy. Antioxidant activity was evaluated using DPPH and ABTS radical scavenging assays. Anti-inflammatory and antithrombotic effects were assessed through inhibition of platelet aggregation induced by platelet-activating factor (PAF) and adenosine diphosphate (ADP) in human platelets. **Results:** All extracts showed significant antioxidant activity. FTIR and LC–MS analyses confirmed the presence of phenolics, flavonoids, carotenoids, and polar lipids. Kiwi peel extract exhibited the strongest inhibition of PAF- and ADP-induced platelet aggregation, attributed to its higher content of phenolics and unsaturated polar lipids. LC–MS data indicated a favorable fatty acid profile with high omega-9 levels and a low omega-6/omega-3 ratio. Polar lipid structural analysis revealed a predominance of phospholipids with unsaturated fatty acids at the sn-2 position. **Conclusions:** Kiwi by-products are valuable sources of health-promoting bioactives with antioxidant and anti-inflammatory potential. These findings support their incorporation into nutraceutical, nutricosmetic, and cosmeceutical products and lay the groundwork for further studies on safety, efficacy, and practical application.

## 1. Introduction

Kiwi (Actinidia deliciosa) is widely recognized for its high content of bioactive compounds, such as polyphenols, flavonoids, and carotenoids, which contribute to its antioxidant and anti-inflammatory effects. In recent years, there has been growing interest not only in the fruit itself but also in the valorization of its by-products—peels, seeds, and leaves—which are often discarded as waste. These by-products offer a promising source of natural compounds that can be utilized in functional foods, nutraceuticals, and cosmetic formulations, aligning with the principles of sustainability and circular economy [1,2].

Although the beneficial effects of kiwi fruit have been extensively studied, the bioactive potential of its by-products remains less explored. Moreover, while peels, seeds, and leaves of various other fruits have been shown to contain polyphenols, unsaturated fatty acids, and carotenoids, the unique amphiphilic nature of kiwi by-products—containing both hydrophilic and lipophilic compounds—sets them apart. This dual composition may offer enhanced biological activity [1,3,4,5].

Despite extensive studies on the kiwi fruit itself, the bioactivity of its by-products remains relatively underexplored. Emerging research in the fields of circular economy and sustainable agriculture highlights the significance of valorizing all components of the fruit. This approach not only mitigates environmental impact but also unlocks the potential of bioactive compounds present in agri-food waste [4,5,6,7].

To address this gap, the present study aims to evaluate the antioxidant, anti-inflammatory, and antithrombotic potential of kiwi and its by-product extracts, focusing on their amphiphilic composition. Advanced analytical techniques such as liquid chromatography-mass spectrometry (LC-MS) and Fourier-transform infrared (FTIR) spectroscopy were employed to identify the molecular classes of these bioactives. The extracts were then assessed for their capacity to scavenge free radicals and inhibit platelet-activating factor (PAF) and adenosine diphosphate (ADP)-induced effects.

This work highlights, for the first time, the integrated investigation of kiwi by-products using both chemical profiling and functional assays. The findings not only demonstrate the potential of these extracts in health-promoting applications but also support the sustainable utilization of agro-industrial waste, paving the way for the development of novel nutraceutical and cosmeceutical products.

## 2. Materials and Methods

### 2.1. Materials

All solvents that were used for extractions and analyses (methanol, ethanol, n-octane, petroleum ether, and chloroform), as well as reagents used in analyses, comprised Tris(hydroxymethyl)aminomethane (Tris), hydrochloric acid (HCl), sodium carbonate (Na_2_CO_3_), 2,2-diphenyl-1-picrylhydrazyl (DPPH), 2,2′-azino-bis(3-ethylbenzothiazoline-6-sulfonic acid) (ABTS), sodium persulfate, acetic acid, and sodium acetate. Standards for phenolic compounds, specifically gallic acid, catechin, and quercetin, as well as polar lipids from soy, β-carotene, and 6-hydroxy-2,5,7,8-tetramethylchroman-2-carboxylic acid (Trolox), were purchased from Sigma-Aldrich (St. Louis, MO, USA). UV-VIS spectrophotometry was employed for all spectrophotometric analyses, using a uniSPEC 2 spectrophotometer (LLG Labware, Meckenheim, Germany), while structural spectrometric analyses of samples and standards were conducted using attenuated total reflectance Fourier-transform infrared (ATR-FTIR) spectroscopy on a Perkin Elmer Frontier ATR/FT-NIR/MIR spectrometer (PerkinElmer, Waltham, MA, USA).

### 2.2. Extraction of Bioactive Compounds from Juice and By-Products

Extracts were obtained from juice and by-products of organic kiwifruit (*Actinidia deliciosa*), each weighing 100 g, cultivated organically in the region of Macedonia, Greece. Each kiwifruit was thoroughly washed and peeled to separate the pulp from the peel. The pulp of each sample was processed using a juicer to obtain both the juice and undissolved pulp by-products. These by-products, along with the peel, constituted the total by-product fraction. Thus, six total samples were generated for analysis: three juice samples (1, 2, 3) and three by-product samples (4, 5, 6), yielding two samples per fruit. Each sample was placed in pre-labelled beakers for blundering and extractions.

Extractions were performed on each kiwi juice or by-product sample by adding a solvent mixture in a ratio of chloroform/methanol/water (1:2:0.8; *v*/*v*/*v*), where the water percentage reflects the juice content. This procedure created monophasic systems, which after thorough blundering were then filtered to obtain the extracts from precipitated residues. Vacuum filtration was conducted using a Buchner filtration apparatus equipped with filter paper. The filtrates were transferred to a separatory funnel, where additional water and chloroform were added in optimized volumes, achieving phase separation with a final solvent ratio of chloroform/methanol/water set to 1:1:0.9 (*v*/*v*/*v*) based on the Bligh & Dyer [8] methodology, as modified by Vandorou et al. (2025) [9].

Total lipids (TL) extracted from each sample appeared in the lower chloroform phase, which was then collected in round-bottom flasks. Solvent removal was carried out through flash rotary evaporation at 37 °C under vacuum (Buchi Rotavapor, Mason Technology Ltd., Dublin, Ireland). Remaining TLs were re-dissolved in a minimal volume (1–3 mL) of 1:1 (*v*/*v*) chloroform/methanol solution, allowing easy transfer to pre-weighed glass tubes, where solvents were fully evaporated in a nitrogen stream atmosphere. From these TL samples, the more lipophilic compounds (total lipophilic content; TLC) and the more polar amphiphilic compounds (total amphiphilic content; TAC) were separated using the Galanos & Kapoulas [10] counter-current distribution methodology with pre-equilibrated petroleum ether (for obtaining the TLC) and 87% ethanol in water (for obtaining the TAC) biphasic system, as modified by Vandorou et al. (2025) [9].

Solvent evaporation was repeated under vacuum to dry the TLC and TAC extracts, followed by transfer to pre-weighed glass tubes using a minimal amount of petroleum ether for TLC and 1:1 chloroform/methanol solution for TAC. Lipid extracts were then evaporated under a nitrogen stream, weighed to assess extraction yield, and stored at −20 °C (under nitrogen) until further analysis.

### 2.3. Platelet Aggregometry Biological Assays

Bioassays were conducted to assess the anti-platelet and anti-inflammatory activities of bioactive extracts from both juice and by-product samples according to Tsoupras et al. (2025) [11]. Each extract’s inhibitory effects on human platelet aggregation were assessed on human platelet-rich plasma (hPRP) of healthy donors. Aggregation was induced in hPRP by both the inflammatory and thrombotic mediator platelet-activating factor (PAF) and the well-established standard platelet agonist adenosine diphosphate (ADP) in the absence and presence of bioactive extracts for comparisons.

The anti-inflammatory and antithrombotic potency of each sample was expressed as the half-maximal inhibitory concentration (IC_50_) ± standard deviation (SD), measured as the mass (µg) of bioactive extract per aggregometer cuvette required to inhibit PAF- or ADP-induced platelet aggregation by 50%.This approach provides a quantifiable metric for comparing the efficacy of each bioactive lipid extract in inhibiting platelet activation and aggregation induced by two distinct pathways associated with inflammatory (PAF) and/or thrombotic events (ADP).

### 2.4. FT-IR Based Structural Analysis of TAC Extracts from Lemna Minor and Nelumbo Nucifera

The ATR-FTIR was performed as previously described [12]. The spectrum obtained for each sample was analyzed against a set of standard samples for which the same procedure was previously performed. These standard samples are quercetin, catechin, gallic acid, beta-carotene, and polar lipids from soy. At the same time, the spectrum of isopropanol was also analyzed in order to reduce any error in the study that may be derived due to solvent interference in the sample under study.

### 2.5. Fatty Acid Composition and Polar Lipids’ Structural Analysis by Liquid Chromatography-Mass Spectrometry (LC-MS)

The fatty acid composition and the structural analysis of the polar lipids (PL) present in the TAC extracts from both kiwifruit juice and by-product samples was assessed using liquid chromatography-mass spectrometry (LC-MS), as previously described by Vandorou et al. [9]. Briefly, dried TAC extracts were re-dissolved in 500 µL of a dichloromethane/methanol solution (1:2, *v*/*v*) and centrifuged for 6 min at 13,000 rpm (Heraeus Biofuge Stratos, Fisher Scientific Ltd., Dublin, Ireland). The supernatants were filtered through 3 kDa ultrafiltration membranes (Amicon Ultra 3 k, Merck Millipore Ltd., Darmstadt, Germany) to ensure removal of high-molecular-weight impurities. To obtain fatty acid profiles, 10 µL of each filtrate was injected into an HPLC system (Agilent 1260 series, Agilent Technologies Ireland Ltd., Cork, Ireland) coupled with a quadrupole time-of-flight mass spectrometer (Agilent 6520) using electrospray ionization (ESI) in negative mode. Separation was achieved on an Agilent C18 Poroshell 120 column (2.7 µm, 3.0 × 150 mm) under a gradient elution. The mobile phase consisted of 2 mM ammonium acetate in water (Phase A) and 2 mM ammonium acetate in 95% acetonitrile (Phase B). The flow rate was set at 0.3 mL/min for the initial 5 min, gradually increasing to 0.6 mL/min after 10 min and maintained until completion of the run.

Mass spectrometric detection ranged from *m*/*z* 50 to 1100, with reference masses of 1033.988 and 112.9855 for negative ionization mode. Capillary voltage was set at 3500 V, with skimmer and fragment or voltages at 65 V and 175 V, respectively. Drying gas flow rate, pressure, and nebulizer temperature were maintained at 5 L/min, 30 psi, and 325 °C. Method validation was performed by comparing retention times and *m*/*z* values with known standards, including several saturated and unsaturated fatty acids: lauric (C12:0), myristic (C14:0), palmitic (C16:0), stearic (C18:0), oleic (C18:1n-9), linoleic (C18:2n-6), and alpha-linolenic (C18:3n-3), among others. The relative abundance of each identified fatty acid in lipid extracts was determined as an average of three replicates. Due to the nature of peak areas, these relative values are semi-quantitative and should be interpreted accordingly.

The assignment of fatty acids and PL species was based upon a combination of survey, daughter, precursor and neutral loss scans, and the identity of the bioactive PL molecules was verified using the LIPID MAPS: Nature Lipidomics Gateway (www.lipidmaps.org, accessed on 20 February 2025) by using the lowest delta values combined with the results obtained from the LC-MS analysis on the fatty acid composition of the saponified PL, as previously described by Vandorou et al. [9].

### 2.6. Assessment of Total Phenolic Content, Carotenoid Content, and Antioxidant Activities

#### 2.6.1. Sample Preparation for Further Analysis

For subsequent analyses, each dried sample of TAC was reconstituted in 1 mL of ethanol, while TLC was dissolved in 1 mL of octane. Each solution was divided into aliquots for quantifying phenolic and carotenoid contents, as well as for bioactivity assessments, with further solvent evaporation performed to produce dried TPC and TLC samples for each aliquot used in each analytical method.

#### 2.6.2. Total Phenolic Content (TPC) Analysis

The TPC of TAC and TLC extracts from juice and by-product samples was measured using the Folin–Ciocalteu method, as described by [13]. Briefly, 1 mL of deionized water and 1 mL of Folin–Ciocalteu reagent were added to each sample. After a 7 min incubation, 3 mL of Na_2_CO_3_ solution was added, followed by a 2 h dark incubation. During reagent additions and every 30 min during incubation, samples were vortexed to ensure uniform mixing. Absorbance was measured at 765 nm, and TPC was quantified based on a gallic acid standard curve, with results expressed as mg gallic acid equivalents (GAE) per gram of dry weight (DW) of each extract.

#### 2.6.3. Total Antioxidant Activity (TAA) Evaluation

The antioxidant activity of each sample was evaluated using the DPPH, ABTS, and FRAP assays, following methodologies as described by Papadopoulou et al. [13].

DPPH Assay: Each sample was mixed with 0.2 mL ethanol, 0.8 mL Tris-HCl buffer (pH 7.4), and 1 mL DPPH solution. After each reagent addition, samples were vortexed and incubated at room temperature for 30 min. Absorbance was recorded at 517 nm, and inhibition (%) was calculated as follows:

Inhibition (%) = ((A_1_ − A_2_)/A_1_) × 100

where A_1_ is the control absorbance and A_2_ is the test sample absorbance. The IC_50_ concentration required to inhibit DPPH radicals by 50% was calculated and expressed in Trolox equivalents (TEAC).

TEAC = IC_50_ of Trolox (μg/L)/IC_50_ of the sample (μg/L).

ABTS Assay: For each sample, 2 mL of ABTS solution was added, vortexed, and incubated in darkness for 7 min before measuring absorbance at 734 nm. Trolox served as the standard, with results expressed as µmol TE/g dry weight (DW), using the equation:

ABTS (μmolTE/gDW)= (c × V × t)/m

where c is the Trolox concentration from the standard curve, V the sample volume (mL), t the dilution factor, and m the sample dry weight (g).

FRAP Assay: Each sample received 3 mL of FRAP reagent, was vortexed, and incubated at 37 °C in darkness for 15 min. Absorbance was recorded at 593 nm, with results in µmol TE/g DW as per the equation above.

#### 2.6.4. Total Carotenoid Content (TCC) Analysis

The TCC of each extract was quantified based on the method as described by Papadopoulou et al. [11]. Each sample was dissolved in 2 mL of octane, and absorbance was measured at 450 nm. Carotenoid concentration was calculated using a β-carotene standard curve, with results expressed in mg β-carotene equivalents (CE) per gram of DW of each extract.

#### 2.6.5. Statistical Analysis

All bioactivity assays, including antioxidant and platelet aggregation inhibition assays, were conducted using three independent biological replicates (*n* = 3). For the anti-inflammatory and antiplatelet activities, each of the 3 extracts was assessed in different blood samples from several individual healthy donors (N = 9 quantifications in total for each extract). Prior to analysis, data normality was assessed using the Kolmogorov–Smirnov test. For datasets that followed a normal distribution (IC_50_ values of the anti-inflammatory and antiplatelet effects, as well as fatty acid content), one-way analysis of variance (ANOVA) was applied, followed by LSD post hoc test for multiple comparisons. Results in this case are presented as mean values ± standard deviation (SD). In cases where the data did not meet the assumptions of normality (phenolic and carotenoid contents and antioxidant activities), the non-parametric Kruskal–Wallis test was used. Results in this case are presented as min, max, and median. Statistical comparisons were made between different sample types (e.g., juice vs. by-products) and between extraction methods (TAC vs. TLC). Statistical significance was set at *p* < 0.05.

## 3. Results

### 3.1. Yield of Extraction

According to Table 1, significant differences are observed in the yields of total lipids (TL), TAC, and TLC extracts from kiwi juice and its by-products. The extracted TAC from the by-product were found to be (0.249–0.263) g of TAC per 100 g of by-product sample, almost an order of magnitude higher than what was extracted from the juice, since the extracted TAC from the juicewere found to be approximately (0.030–0.032) g per 100 g of juice sample. This indicates that the by-product is a richer source in TAC bioactive compounds, suggesting it may be more suitable for extraction of such ingredients for potential industrial use. Regarding TLC, the juice shows a concentration of approximately (0.045–0.066) g per 100 g of sample, while the by-product contains a five times higher concentration of approximately (0.107–0.322) g per 100 g of sample. Although there is a larger variability in the by-product, and, similar to what was observed in the TAC extracts, the increased lipophilic content indicates a richer presence of lipid-soluble substances in the by-products compared to the juice. Subsequently, when comparing the yield of TL, the by-product shows a much higher concentration (0.356–0.584) g per 100 g of sample) than the juice (0.074–0.098) g per 100 g of sample). This disparity indicates that the by-products—comprising peels and other residues—are substantially richer in amphiphilic and lipophilic compounds, which are either insoluble or only sparingly soluble in water. During juice extraction, these bioactive constituents tend to become sequestered within the fiber-rich matrix of the by-product. This observation aligns with previous studies on other health-promoting fruits, such as organically grown apples and avocados, where by-products yielded significantly higher concentrations of both lipophilic and, in particular, amphiphilic compounds compared to their corresponding juices [9,14].

In summary, kiwi by-products exhibit a higher content of both amphiphilic compounds and total lipids, underscoring their potential as a valuable source for bioactive compound recovery. In contrast, the juice contains a moderate concentration of lipophilic compounds, which may limit its applicability in certain contexts. These findings suggest that kiwi by-products offer greater value for applications extending beyond conventional juice consumption.

### 3.2. Total Phenolic and Carotenoid Content of Kiwi and Its By-Products

The analysis of kiwi extracts reveals distinct differences in the content and stability of bioactive compounds between kiwi juice and kiwi by-products, as well as comparisons to existing literature on kiwi peel and pulp. Both the total carotenoid content (TCC) and total phenolic content (TPC) of the extracts amphiphilic, lipophilic, and total lipid content (TLC, TAC, and TL, respectively) were assessed. As shown in Table 2, similar values of TCC were observed for the TLC extracts from both kiwi juice (1.87–3.41 CE)and by-products, while kiwi juice TAC showed statistically significantly higher TCC (1.91–6.77 CE) than that of the TAC extracts from kiwi by-products (0.73–1.60 CE) (*p* < 0.05). Subsequently, kiwi juice TL also showed statistically significantly higher TCC (3.78–9.59 CE) than that of the TL extracts from kiwi by-products (2.62–3.07 CE) (*p* < 0.05), which is consistent with the so-far reported outcomes for kiwi [15]. Nevertheless, the obtained TCC in the TL extracts of kiwi by-products are comparable to those previously reported for extracts from kiwi peel and pulp (5.70 mg/g DW for peel and 1.55 mg/g DW for pulp).

In contrast, as shown in Table 3, the TPC for the TL (23.33–55.75 GAE), TAC (6.74–48.69 GAE), and TLC (7.06–16.59 GAE) extracts from the juice did not differ from that of the kiwi by-products (TL: 31.89–41.55 GAE, TAC: 18.42–25.61 GAE, and TLC: 11.39–22.45 GAE, respectively), indicating that the by-products are also a good sustainable source for obtaining extracts rich in phenolic compounds. This is also supported by the fact that the TPC detected in all extracts from the kiwi by-products is much higher than those reported in the literature, where the phenolic content of kiwi peel and pulp has previously been reported to be 5.70 mg GAE/g DW for peel and 1.55 mg GAE/g DW for pulp [16], highlighting the successful extraction process of the present study. Similarly, the phenolic content of the juice extracts in this study were also higher than previously reported (1.80–2.20 mg GAE per g of DW [17]), which further supports the effectiveness of the extraction process performed in the present study.

### 3.3. Antioxidant Activities

The antioxidant capacity of the TAC, TLC, and TL extracts from kiwi juice and its by-products were evaluated using the ABTS and DPPH assays, and the results are expressed in ABTS values (μmol of Trolox equivalent (TE)/g DW) and Trolox-equivalent antioxidant capacity (TEAC), respectively, as presented in Table 4 and Table 5.All antioxidant results were statistically analyzed using one-way ANOVA or Kruskal–Wallis tests, followed by Tukey’s or Dunn’s post hoc comparisons, respectively, as described in Section 2.6.5. Statistically significant differences between groups are reported where *p* < 0.05. Kiwi juice TAC extracts showed higher antioxidant capacity with ABTS values of 4.48–13.4 μmol of TE/g DW, compared to that observed for the TAC extracts from kiwi by-products with ABTS values of 2.17–2.93 μmol of TE/g DW (*p* < 0.05). These results indicate that some more polar amphiphilic antioxidants have migrated more towards the water-based kiwi juice rather than remaining in the by-products during juice preparation. In contrast, the ABTS values and thus the antioxidant capacity of the kiwi juice TLC extracts (1.08–2.56 μmol of TE/g DW) did not differ from those of the kiwi by-products TLC (1.58–16.37 μmol of TE/g DW). Subsequently, the ABTS values of the kiwi juice TL extracts (5.62–16.2 μmol of TE/g DW) also did not differ from those of the kiwi by-products TL extracts (3.75–19.25 μmol of TE/g DW), respectively. Bibliography data for such extracts from kiwi with various methodologies align with these findings, as kiwi by-products have previously been found to exhibit ABTS values up to 5.70 μmol Trolox/g [16,18,19], further confirming the antioxidant potency of kiwi by-product extracts.

Interestingly, by performing the DPPH assay in all these extracts, similar outcomes were obtained as shown in Table 5. More specifically, kiwi juice TAC extracts showed higher antioxidant capacity with TEAC values of 0.0014–0.0015, compared to that observed for the TAC extracts from kiwi by-products with TEAC values of 0.0007–0.0013 (*p* < 0.05). Again, these results support the notion that some more polar amphiphilic antioxidants migrate towards the water-based kiwi juice more rather than remaining in the by-products during the making of the juice at the juicer. In contrast, the TEAC values and thus the antioxidant capacity of the kiwi juice TLC extracts did not differ from those of the kiwi by-products TLC. Subsequently, the TEAC values of the kiwi juice TL extracts also did not differ from those of the kiwi by-products TL extracts, respectively, which further indicates that extracts from kiwi by-products possess potent antioxidant capacity. The literature supports this observation, reporting low IC_50_ values and thus high TEAC values for kiwi seeds and peel, confirming the antioxidant efficacy of these by-product-derived compounds [20].

In conclusion, the obtained results from both the DPPH- and ABTS-based antioxidant assays extracts rich in phenolics and carotenoids like those obtained from kiwi by-products, can be valorized as bioactive ingredients in several applications of functional products (i.e., functional foods, supplements, nutraceuticals, cosmetics), with antioxidant health-promoting properties against oxidative stress and associated pathologies.

### 3.4. FT-IR

The spectral analysis of the kiwi sample revealed key peaks aligning with compounds in the reference standards, such as phytochemicals including simple phenolics such as gallic acid, more complex phenolics such as the flavonoids catechin and quercetin, but also oilier amphiphilic bioactives such as soy polar lipids and β-carotene. As shown in Table 6, a broad peak observed in the 3200–3600 cm^−1^ region suggests the presence of hydroxyl groups (-OH), consistent with signals seen in catechin, quercetin, and gallic acid standards, indicating phenolic compounds. Peaks in the 2850–3000 cm^−1^ range, linked to C-H stretching, suggest fatty acids or hydrocarbons, resembling those found in polar lipids, but also hydrocarbons found in carotenoids. The strong peak in the 1700–1750 cm^−1^ region indicates carbonyl groups (C=O), similar to the standards of gallic acid and polar lipids, suggesting esters or carboxylic acids. Peaks between 1600–1400 cm^−1^, associated with aromatic C=C stretching, point to flavonoid content, matching catechin and quercetin structures. Further, the 1200–1000 cm^−1^ range shows C-O stretching peaks, indicative of alcohols, ethers, or esters, correlating with signals from catechin, gallic acid, and quercetin standards. The fingerprint region (600–1500 cm^−1^) confirms the presence of similar functional groups seen in gallic acid, polar lipids, and quercetin.

These findings align with the literature, which identifies phenolic compounds, such as catechin, chlorogenic acid, and quercetin, in kiwi fruit. The detection of hydroxyl and aromatic groups supports the presence of flavonoids such as quercetin and catechin, while the carbonyl groups suggest lipids and esters. Overall, the kiwi sample contains a rich array of phenolic compounds, lipids, and carotenoids, highlighting its potential for nutraceutical applications [4,6,21].

### 3.5. LC-MSAnalysis of the TAC Extracts from Kiwi Juice and Its By-Products—Fatty Acid Composition and Structural Elucidation of Polar Lipids

As shown in Table 7 several differences were observed in the fatty acid composition of polar lipids (PL) present in the TAC extracts from kiwi juice to those obtained for the PL of TAC from its by-products. The unsaturated fatty acids (UFA content of the PL in the TAC extracts from the juice (70.69 ± 0.09) was found to be higher than that of PL of the TAC extracts from the by-products (45.32 ± 4.231). More specifically, the monounsaturated fatty acids (MUFA) content was higher in the PL of the TAC from the juice (31.94 ± 0.05%) than that of the PL of the by-products (20.70 ± 0.52%), with oleic acid being the primary contributor (30.00 ± 0.03% in juice), indicating the enhanced nutritional value of the TAC extracts due to such MUFA content. The polyunsaturated fatty acids (PUFA) content of PL in both extracts also followed a similar pattern, with the PL of the TAC from the juice containing higher levels (38.75 ± 0.05%) compared to the PL from the by-products (24.62 ± 4.11%). The n-3 PUFA, especially alpha-linolenic acid (ALA), was notably higher in the PL from the juice (28.91 ± 0.07%), highlighting its advantages in reducing inflammation and improving heart health. Nevertheless, the n-6/n-3 ratio was similarly favorably low in the PL from both the juice (0.34) and its by-products (0.51), which further support an anti-inflammatory and cardio-protective potential for the TAC extracts, either from the kiwi juice or from its by-products, since the lower the value from 15/1 for this ratio the higher the preventative and therapeutic potential for a product containing these PUFA [22] (in this case the PL of the TAC extracts from kiwi juice and its by-products).

Conversely, the PL of the TAC from the juice showed lower saturated fatty acids (SFA) content (29.31 ± 0.09) compared to that of the PL in the TAC of the by-products (54.68 ± 4.23%), with palmitic acid (C16:0) and stearic acid (C18:0) being the dominant SFAs for the PL of the juice, while stearic acid was significantly more abundant in the PL of the by-products (30.21 ± 2.07%) in comparison to the content for this fatty acid found in the PL of the juice (10.20 ± 0.14%).

These experimental findings are partially consistent with the bibliographic data, which highlight that kiwi by-products, particularly the seeds and peel, are rich in unsaturated fatty acids, especially linoleic acid (n-6) and alpha-linolenic acid (ALA, n-3). In the current study, however, the PL fraction of the TAC extract from the juice exhibited a notably higher ALA content (28.91%) compared to that of the by-products, potentially reflecting a greater concentration of membrane-bound lipids in the juice extract. Furthermore, kiwi seed oil contains up to 35% totallipids, with a predominance of polyunsaturated fatty acids, particularly linoleic and linolenic acids, which may explain the moderate PUFA content observed in the PL of by-product extracts. Importantly, the low n-6/n-3 PUFA ratios observed in both sources (0.34 for juice and 0.51 for by-products) align with the literature’s emphasis on the anti-inflammatory and cardioprotective potential of such lipid profiles, since values below 4:1 are associated with reduced chronic inflammation and cardiovascular risk [1].

Similar differences in fatty acid composition and bioactivity have been observed in other fruit by-products such as apple and avocado, supporting the relevance of our findings. Apple pomace PLs predominantly contain saturated fatty acids, with palmitic acid (C16:0) and stearic acid (C18:0) being the most abundant. However, they also possess appreciable amounts of polyunsaturated fatty acids, including linoleic acid (LA, C18:2n-6) and ALA, with LA being the dominant PUFA. In contrast, avocado-derived PLs are characterized by a high content of monounsaturated fatty acids (MUFAs), primarily oleic acid (C18:1n-9), which is renowned for its cardioprotective and anti-inflammatory effects. Additionally, avocado extracts exhibit higher total phenolic content and carotenoids, contributing to their enhanced antioxidant capacity. Unlike kiwi juice, neither avocado nor apple extracts reached comparable n-3 PUFA levels or n-6/n-3 ratios, indicating that kiwi may provide superior omega-3 benefits, while avocado offers stronger MUFA-driven bioactivity, and apple delivers a broad-spectrum lipid functionality suitable for anti-inflammatory formulations [9,14].

The comparative analysis of fatty acid profiles in unsaponified TAC extracts from kiwifruit juice and by-products also revealed the presence of some amounts of free fatty acids (FFA) within the TAC extracts, while again distinct differences were observed in the obtained SFA, MUFA, and PUFA content of these FFA of the TAC from the kiwifruit juice compared to these contents for the FFA of the TAC from the by-products (Table 7). The FFA content of the TAC from the juice exhibited a higher content of SFA (92.89 ± 0.06%) compared to the relative content for the FFA of the TAC from the by-products (87.91 ± 0.08%), and especially when compared to the aforementioned SFA content of the PL from both sources. The FFA of the TAC extracts from the by-products was richer in UFA, with a concentration of 12.09 ± 0.08%, versus 7.11 ± 0.06% observed for the UFA of the FFA of the TAC from the juice, but both values were still significantly lower than the UFA content of the PL present in these TAC extracts. Nevertheless, the MUFA levels of the FFA were also higher in the by-products (9.78 ± 0.05%) compared to that of the FFA in the TAC of the juice (5.62 ± 0.10%, with oleic acid (C18:1), a key MUFA, being more concentrated in the FFA of the TAC from the by-products (9.23 ± 0.08%) than that from the juice (5.06 ± 0.09%). Similarly, even though PUFAs were in low levels in both FFA contents, still those present at the FFA of the by-products (2.32 ± 0.03%) were higher compared to those from the juice (1.49 ± 0.05%), further supporting a contribution of the UFA of the FFA of the TAC extracts from the by-products to a potential link with anti-inflammatory and cardiovascular health benefits.

Regarding the LC-MS-based structural analysis of phospholipids (PL) in the TAC extracts from both kiwi juice and by-products, survey scans conducted in the negative ion mode within the *m*/*z* range of 600–1000 revealed that the predominant phospholipid classes were glycerol-based (GP, glycerophospholipids), particularly various molecular species of phosphatidylcholines (PC) and phosphatidylethanolamines (PE). Additionally, glycerolipid classes were detected and identified as di(acyl/alkyl)glycerols (DG).

LC-MS analysis of the TAC extracts from kiwi juice and by-products revealed several classes of polar lipid bioactives, primarily diacylglycerols (DG), phosphatidylcholines (PC), and phosphatidylethanolamines (PE). These molecular species were formed through various combinations of eight key fatty acids, including palmitic, oleic, linoleic, and alpha-linolenic acids (Table 8. Notably, juice-derived PLs contained higher levels of unsaturated fatty acids—especially omega-3-rich alpha-linolenic acid—while by-products presented more diversity in DG species. The identified polar lipids—diacylglycerols (DG), phosphatidylcholines (PC), and phosphatidylethanolamines (PE)—are known for their distinct bioactive roles, including anti-inflammatory, metabolic, and neuroprotective functions, as supported by prior studies. In our study, the structural profiling of these PLs in kiwi extracts provides novel evidence of their presence in both juice and by-products [23,24].

When it comes to PC, it’s highly anti-inflammatory since it inhibits proinflammatory cytokines and oxidative tissue damage that results in diseases like neurodegeneration and systemic inflammation. Furthermore, PC plays an important role in maintaining synaptic function and neuroplasticity, which are crucial for memory formation and cognitive performance. Experiments also proved that PC could improve gut barrier function, restructure the gut microbiota, and induce short-chain-fatty-acid production, which would further regulate the gut–brain axis. According to some literature sources, phosphatidylcholines were proposed as markers for accessing omega-3 status in the organism due to their positive effects on the cardiovascular system [25,26].

In addition, phosphatidylethanolamine (PE) was unveiled as an effective compound facilitating autophagy, a key process to cellular homeostasis and longevity, in carrying the longevity signal in yeast, worms, and even flies. It also increases resistance to oxidative stress, delays age-associated skeletal muscle atrophy, and protects from beta-amyloid-induced toxicity, therefore suggesting potential protective roles in neurodegenerative diseases such as Alzheimer’s disease. Moreover, it plays a role in mitochondria through its participation in maintaining the membrane, respiration, and protein-folding competence; therefore, its influence may extend to overall cellular health, anti-aging, and disease prevention [27,28,29].

All the above, suggest that these PLs located in kiwi have multiple assets surrounding health, ameliorating metabolic health, brain work, etc., making kiwi a healthy food source and its by-products a sustainable source of these bioactives.

### 3.6. Anti-Inflammatory and Anti-Platelet Properties of Kiwi

PAF is a potent inflammatory mediator implicated in several inflammation-related pathological situations (Tsoupras et al.) [30]. PAF exerts its distinct effects by binding on a specific G-protein coupled membrane receptor (PAFR) that is present in all cells of our body, including platelets. Thus, an effective cell-model that allows the quantification of the anti-inflammatory anti-PAF inhibitory effects of a bioactive compound/extract/drug against the PAF-related pathway is by assessing the ability of this bioactive to inhibit PAF-induced aggregation of platelets in the presence of a range of concentrations for this bioactive, from which the half maximum inhibitory activity (IC_50_ value) for this bioactive can be obtained as per [31]. The IC_50_ for a bioactive extract against the PAF pathway in platelets provides the strength of its inhibitory effect against this inflammatory mediator, since the lower the IC_50_ value of an extract, the stronger its anti-PAF activities. In the present study the anti-PAF anti-inflammatory effects of the TAC and TLC extracts from both the kiwifruit juice and its by-products were evaluated in human platelet-rich plasma (PRP) by quantifying their IC_50_ values against PAF-induced aggregation of PRP, in comparison to their effects against a standard platelet agonist, ADP, which also induces platelet aggregation but through a different thrombotic pathway. Statistical analysis of IC_50_ values revealed significant differences among the extracts (*p* < 0.05).

**Figure 1:**
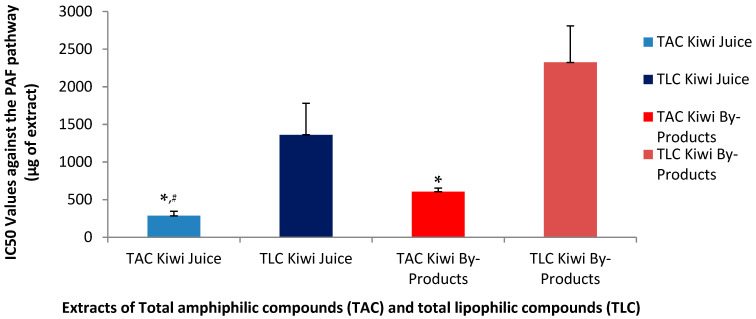
Anti-inflammatory effects of TAC and TLC extracts from kiwifruit juice and by-products against a potent mediator of inflammation (PAF). Results are expressed as IC_50_ values (half maximum inhibitory activity) against the aggregation of PRP induced by the thrombo-inflammatory PAF-associated pathways. * Indicates a statistically significant difference (*p* < 0.05) of the anti-PAF effects of TACs compared to those of their TLCs, # indicates a statistically significant difference (*p* < 0.05) of the anti-PAF effects of TACs from the juice compared to that of the TACs from the by-products. (TAC = total amphiphilic content; TLC = total lipophilic content; PAF = platelet-activating factor; PRP = plasma rich platelets).

**Figure 2:**
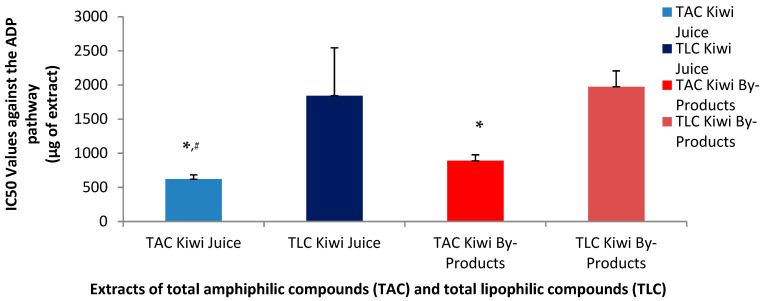
Anti-platelet effects of TAC and TLC extracts from kiwifruit juice and by-products against the thrombotic pathway of a standard platelet agonist (ADP). Results are expressed as IC_50_ values (half maximum inhibitory activity) against the ADP-induced aggregation of PRP. * Indicates a statistically significant difference (*p* < 0.05) of the anti-ADP effects of TACs compared to those of their TLCs, # indicates a statistically significant difference (*p* < 0.05) of the anti-ADP effects of TACs from the juice compared to that of the TACs from the by-products. (TAC = total amphiphilic content; TLC = total lipophilic content; PAF = platelet-activating factor; PRP = plasma rich platelets).

As shown in Figure 1 and Figure 2, the inhibitory effects of the TAC extracts from the kiwifruit juice were found to be stronger than those of the TAC extracts from the kiwifruit by-products against both the ADP and PAF pathways, suggesting that the juice TAC extracts contain more potent anti-inflammatory PAF-inhibitors and anti-platelet ADP-inhibitors. Regarding the effects of the TAC and TLC extracts from both sources on the PAF pathway, it was found that in both the juice and the by-products TAC extracts showed statistically significantly higher anti-PAF activities (lower IC_50_ values) than those of the TLC extracts from both the kiwifruit juice and its by-products. Moreover, the TAC from the juice were even more effective than those of the by-products, since the IC_50_ value for TAC in juice had a mean of 286 µg, which is nearly half of the IC_50_ value (606 µg) observed for the TAC of the by-products to inhibit the PAF pathway. Following the same pattern, the mean IC_50_ value for the TLC in juice was 1362 µg, which is much lower compared to 2325 µg for the TLC of the by-products, but both TLC extracts still showed very low activities against PAF compared to the aforementioned activities of the TAC extracts for each source.

Concerning the anti-ADP antiplatelet effects of the TAC and TLC extracts from both the juice and its by-products, the TAC from the juice again demonstrated a greater inhibitory effect against this pathway too, with a mean IC_50_ value at 620 µg, significantly lower than the relative IC_50_ of the TAC from the by-products (890 µg). Similar high IC_50_ values were observed for the TLC from the juice (1842 µg) and from the by-products (1975 µg) to inhibit the ADP-pathway of PRP aggregation.

Although TAC from the kiwifruit by-products inhibited both pathways, their efficacy was lower than that of the TAC from the juice. Notably, the PAF pathway was inhibited more effectively by the TAC from the by-products compared to the ADP pathway, yet still less effectively than by the TAC from the juice. These findings suggest that the amphiphilic bioactive compounds of the TAC in kiwifruit juice are either more potent against both pathways and especially against the PAF-pathway or more readily bioavailable and thus again more active.

The inhibition of the ADP pathway is primarily linked to thrombosis prevention, while the stronger inhibitory effect on the PAF pathway indicates that kiwifruit juice may be more effective in suppressing inflammatory responses. Thus, the lower IC_50_ values observed for both pathways support the hypothesis that the bioactive amphiphilic compounds in the juice are more efficient compared to those found in the by-products.

Although the by-products exhibited a lower inhibitory capacity compared to kiwi juice, they still demonstrated notable potential to inhibit both ADP- and PAF-induced pathways. In particular, the amphiphilic compounds present in the by-products showed greater inhibitory activity against the PAF pathway than the ADP pathway, suggesting a potential for more effective modulation of inflammatory responses. Despite the higher potency of the TAC derived from kiwi juice, the TAC extracted from by-products remains a valuable source of natural inhibitors of inflammatory and hemostatic processes. These findings highlight the potential of kiwi by-products as sustainable resources for the development of dietary supplements and other functional applications, including cosmetics with anti-aging and health-promoting properties.

The stronger inhibitory effects of the TAC extracts derived from kiwifruit juice, as reflected by significantly lower IC_50_ values against both the PAF and ADP pathways, are consistent with previous studies reporting higher bioactivity and bioavailability of juice-derived amphiphilic compounds. According to the review by Moysidou et al. (2024) [1], kiwi juice is known to be rich in water-soluble phenolics and amphiphilic lipids—such as catechins, chlorogenic acid, and α-linolenic acid—which are more readily released during juicing compared to those bound within by-product matrices. These compounds have been associated with potent anti-inflammatory and cardioprotective effects, offering a mechanistic explanation for the observed higher efficacy of the juice TAC extracts [1].

Our findings also align with results from other fruits, such as avocado and apple, where TAC extracts consistently show greater anti-inflammatory and antithrombotic efficacy compared to TLC extracts. These trends underline the importance of amphiphilic compounds—particularly polar lipids and phenolic-lipid conjugates—in modulating platelet activity and inflammatory signaling. In avocado and apple pomace, such compounds include glycolipids and carotenoid esters, which contribute to their potent bioactivity. Although the kiwi by-products did not exhibit comparable levels of glycolipids, their TAC extracts still demonstrated substantial inhibition of both PAF- and ADP-induced pathways. This supports their potential valorization as sustainable, bioactive-rich ingredients for functional product development [9,14].

The by-products of all three fruits—including kiwifruit—still provided notable bioactivity, especially through their TAC fractions. Between them, kiwi by-products showed lower activity, which seems to be related to the lack of glycolipids in the TAC extracts, whereas such important bioactives were present in the TAC from avocado and apple pomace. Nonetheless, the findings of the present study also support the use of the kiwi by-products as sustainable and cost-effective sources of functional bioactives, suitable for valorization in food, cosmetic, or nutraceutical formulations.

## 4. Conclusions

This study highlights the significant potential of kiwi fruit and its by-products—particularly peels, seeds, and leaves—as rich sources of bioactive compounds with antioxidant, anti-inflammatory, and antithrombotic properties. Through advanced analytical and bioassay methods, we demonstrated that amphiphilic and lipophilic extracts, especially from kiwi by-products, contain high levels of polyphenols, carotenoids, MUFAs and PUFAs (notably oleic acid and omega-3 fatty acids), which are well-established contributors to human health. The higher yield and bioactivity of by-product extracts support their valorization in functional applications, offering a sustainable approach to reduce agricultural waste.

A key strength of this work lies in its comprehensive molecular and biofunctional profiling of kiwi-derived extracts, particularly focusing on amphiphilic lipids—an area less explored in previous studies. Furthermore, the scalability of this work offers clear industrial potential. The extraction methods used are compatible with sustainable and food-grade processing techniques, making the integration of kiwi by-products into functional food, nutraceutical, or cosmetic formulations feasible. Nevertheless, further work is required to optimize extraction yield, ensure compound stability, and assess economic viability for large-scale production.

While our study provides promising evidence of antioxidant and anti-inflammatory activities of kiwi-derived bioactives, it does not assess their potential cytotoxicity or safety profiles. These aspects are crucial for the development of nutraceuticals or cosmeceuticals. Although the literature indicates that kiwi fruit and its components are generally regarded as safe [1], individual compounds, especially when concentrated or extracted, may elicit unforeseen effects. Future studies should include cytotoxicity screening in relevant human cell lines (e.g., keratinocytes, hepatocytes), skin irritation models, and in vivo safety assays to fully support product development.

Although our findings demonstrate potent antioxidant and anti-inflammatory properties of kiwi extracts in vitro, these assays do not fully replicate the complexity of in vivo conditions. Factors such as absorption, metabolism, distribution, and cellular uptake of these bioactives can significantly influence their physiological activity. Therefore, further studies involving cell-based models and animal or clinical studies are necessary to validate the biological relevance and therapeutic potential of these compounds. Moreover, challenges such as allergenic risks and degradation of active compounds during processing require innovative approaches in extraction and safety assessments. Future research should also prioritize cytotoxicity tests, long-term stability, and sustainability to fully harness the benefits of kiwi bioactives.

## Figures and Tables

**Table 1:** Yields of extraction for TAC, TLC, and TL extracts from juice and kiwi by-products.

Yield for Kiwi Extracts	Max	Min	Median
TAC% (g/100 mL of juice)	0.032	0.030	0.030
TLC% (g/100 mL of juice)	0.066	0.045	0.066
TL% (g/100 mL of juice)	0.098	0.074	0.096
TAC% (g/100 g of by-product)	0.263	0.249	0.250
TLC% (g/100 g of by-product)	0.322	0.107	0.166
TL%(g/100 g of by-product)	0.584	0.356	0.416

TAC = total amphiphilic compounds, TLC = total lipophilic compounds, TL = total lipids.

**Table 2:** Total Carotenoid Content (TCC) of TAC, TLC, and TL extracts from kiwi juice and by-products (results are expressed as mg of β-carotene equivalent (CE) per g of DW of the extract).

Juice			By-Product		
TCC of TAC	TCC of TLC	TCC of TL	TCC of TAC	TCC of TLC	TCC of TL
**Median**	**Median**	**Median**	**Median**	**Median**	**Median**
1.96	2.82	5.37	1.54	1.17	2.77
**Max**	**Max**	**Max**	**Max**	**Max**	**Max**
6.77	3.41	9.59	1.60	2.34	3.07
**Min**	**Min**	**Min**	**Min**	**Min**	**Min**
1.91	1.87	3.78	0.73	1.08	2.62

TAC = total amphiphilic compounds, TLC = total lipophilic compounds, TL = total lipids, DW = dry weight.

**Table 3:** Total Phenolic Content (TPC) of TAC, TLC, and TL extracts from kiwi juice and by-products (results are expressed as mg of gallic acid equivalent (GAE) per g of DW of the extract).

Juice			By-Product		
TPC of TAC	TPC of TLC	TPC of TL	TPC of TAC	TPC of TLC	TPC of TL
**Median**	**Median**	**Median**	**Median**	**Median**	**Median**
29.26	15.24	44.50	20.51	17.91	40.88
**Max**	**Max**	**Max**	**Max**	**Max**	**Max**
48.69	16.59	55.75	25.61	22.45	43.51
**Min**	**Min**	**Min**	**Min**	**Min**	**Min**
6.74	7.06	23.33	18.42	11.39	31.89

TAC = total amphiphilic compounds, TLC = total lipophilic compounds, TL = total lipids, DW = dry weight.

**Table 4:** Antioxidant capacity (ABTS values) of kiwi juice and by-products. Results are expressed as μmol of Trolox Equivalent (TE)/g DW.

	ABTS Values		
Juice	Median	Max	Min
**TAC**	4.54	13.64	4.48
**TLC**	1.81	2.56	1.08
**TL**	6.29	16.20	5.62
**By-product**	**Median**	**Max**	**Min**
**TAC**	2.88	2.93	2.17
**TLC**	5.97	16.37	1.58
**TL**	8.90	19.25	3.75

TAC = total amphiphilic compounds, TLC = total lipophilic compounds, TL = total lipids, DW = dry weight.

**Table 5:** Antioxidant capacity of kiwi juice and by-products. Results are expressed as DPPH-based Trolox Equivalent antioxidant capacity (TEAC) values.

	TEAC Values		
Juice	Median	Max	Min
**TAC**	0.0015	0.0015	0.0014
**TLC**	0.0002	0.0008	0.0002
**TL**	0.0017	0.0022	0.0002
**By-product**	**Median**	**Max**	**Min**
**TAC**	0.0010	0.0013	0.0007
**TLC**	0.0015	0.0017	0.0003
**TL**	0.0015	0.0030	0.0010

TAC = total amphiphilic compounds, TLC = total lipophilic compounds, TL = total lipids.

**Table 6:** Characteristic peaks of kiwi (juice and by-product) sample in ATR-FTIR.

Compounds	β-Carotene	Catechin	Gallic Acid	Polar Lipid	Quercetin
**Peak (cm^−1^)**	1644	3600–3100	3400–2800	3550–3100	3600–3050
**Functional Groups**	**C=C alkene**	**O-H alcohol**	**COOH carboxylic acid**	**O-H alcohol**	**O-H alcohol**
**Peak (cm^−1^)**		1630–1600	1610	2860	1670
**Functional Groups**		**C=C aromatic,** **C=C alkene**	**C=C aromatic**	**C H alkane**	**C=C alkane,** **C=O ketone**
**Peak (cm^−1^)**		1450		1635	1450
**Functional Groups**		**C=C aromatic**		**C=C alkene, C=O ketone**	**C=C aromatic**
**Peak (cm^−1^)**		814 (Fingerprint region)		1455	950, 814,640 (Fingerprint region)
**Functional Groups**				**C=C aromatic**	
**Peak** **(cm^−1^)**				822 (Fingerprint region)	

**Table 7:** The fatty acid profile of PL and FFA in the TAC extracts from kiwifruit juice and its by-products. Fatty acid content of PL was obtained after saponifying PL, while the content of FFA was obtained without saponification of the TAC extracts. Both contents are expressed for each fatty acid (FA) as the percentage composition of the total fatty acids in each evaluated sample (mean ± standard deviation (SD), *n* = 3).

FreeFattyAcids (FFA) Obtained Without Saponification of TAC Extracts				FattyAcids of PL, Obtained After Saponification of TAC Extracts			
Emperical Name	Name for FYP Tables	Juice	By-Products	Emperical Name	Name for FYP Tables	Juice	By-Products
**Caprylic**	**C8:0**	**ND**	**ND**	**Caprylic**	**C8:0**	ND	0.06 ± 0.004
**Pelargonic**	**C9:0**	0.39 ± 0.05	0.34 ± 0.01	**Pelargonic**	**C9:0**	0.04 ± 0.00	0.15 ± 0.013
				**Capric**	**C10:0**	ND	ND
**Lauric**	**C12:0**	0.38 ± 0.01	0.00 ± 0.00	**Lauric**	**C12:0**	0.06 ± 0.01	ND
**Tridecylic**	**C13:0**	0.00 ± 0.00	0.00 ± 0.00	**Tridecylic**	**C13:0**	ND	0.02 ± 0.002
**Myristic**	**C14:0**	0.96 ± 0.06	0.85 ± 0.06	**Myristic**	**C14:0**	0.33 ± 0.03	0.40 ± 0.041
**Pentadecylic**	**C15:0**	0.00 ± 0.00	0.58 ± 0.03	**Pentadecylic**	**C15:0**	0.11 ± 0.02	ND
**Palmitic**	**C16:0**	24.63 ± 1.34	45.83 ± 0.69	**Palmitic**	**C16:0**	18.12 ± 0.14	23.00 ± 2.097
**Palmitoleic**	**C16:1 c9 (n7 MUFA)**	0.56 ± 0.01	0.54 ± 0.03	**Palmitoleic**	**C16:1 c9 (n7 MUFA)**	1.46 ± 0.03	0.64 ± 0.075
**Margaric**	**C17:0**	2.00 ± 0.49	1.43 ± 0.09	**Margaric**	**C17:0**	0.45 ± 0.02	0.86 ± 0.136
**Stearic**	**C18:0**	64.54 ± 1.06	38.87 ± 0.51	**Stearic**	**C18:0**	10.20 ± 0.14	30.21 ± 2.065
**Oleic**	**C18:1 c9 (n9 MUFA)**	5.06 ± 0.09	9.23 ± 0.08	**Oleic**	**C18:1 c9 (n9 MUFA)**	30.00 ± 0.03	19.44 ± 0.525
**Linoleic**	**C18:2 c9,12 (n6 PUFA)**	0.62 ± 0.05	1.04 ± 0.02	**Linoleic**	**C18:2 c9,12 (n6 PUFA)**	9.13 ± 0.03	7.41 ± 0.586
**Linolenic**	**C18:3 c9,12,15 (n3 PUFA)**	0.87 ± 0.06	1.28 ± 0.04	**Linolenic**	**C18:3 c9,12,15 (n3 PUFA)**	28.24 ± 0.04	16.48 ± 4.696
**Stearidonic**	**C18:4 c6,9,12,15 (n3 PUFA)**	ND	ND	**Stearidonic**	**C18:4 c6,9,12,15 (n3 PUFA)**	0.65 ± 0.06	0.30 ± 0.005
**Nonadecylic**	**C19:0**	ND	ND	**Nonadecylic**	**C19:0**	ND	ND
				**Arachidic**	**C20:0**	ND	ND
**Gadoleic**	**C20:1 c9 (n11 MUFA)**	ND	ND	**Gadoleic**	**C20:1 c9 (n11 MUFA)**	0.49 ± 0.01	0.62 ± 0.042
**DihomoLinoleic**	**C18:2 c10,12 (n6 PUFA)**	ND	ND	**DihomoLinoleic**	**C18:2 c10,12 (n6 PUFA)**	0.27 ± 0.01	0.27 ± 0.022
**Dihomolinolenic**	**C20:3 c8,11,14 (n6 PUFA)**	ND	ND	**Dihomolinolenic**	**C20:3 c8,11,14 (n6 PUFA)**	0.43 ± 0.01	0.16 ± 0.005
**Arachidonic**	**C20:4 c5,8,11,14 (n6 PUFA)**	ND	ND	**Arachidonic**	**C20:4 c5,8,11,14 (n6 PUFA)**	ND	ND
**EPA**	**C20:5 c5,8,11,14,17 (n3 PUFA)**	ND	ND	**EPA**	**C20:5 c5,8,11,14,17 (n3 PUFA)**	ND	ND
**Docosadienoic**	**C22:2 c13,16 (n6 PUFA)**	ND	ND	**Docosadienoic**	**C22:2 c13,16 (n6 PUFA)**	ND	ND
**Eranthic**	**C22:3 c5,13,16 (n6 PUFA)**	ND	ND	**Eranthic**	**C22:3 c5,13,16 (n6 PUFA)**	ND	ND
**Adrenic**	**C22:4 c7,10,13,16 (n6 PUFA)**	ND	ND	**Adrenic**	**C22:4 c7,10,13,16 (n6 PUFA)**	ND	ND
**DPA**	**C22:5 c7,10,13,16,19 (n3 PUFA)**	ND	ND	**DPA**	**C22:5 c7,10,13,16,19 (n3 PUFA)**	ND	ND
**DHA**	**C22:6 c4,7,10,13,16,19 (n3 PUFA)**	ND	ND	**DHA**	**C22:6 c4,7,10,13,16,19 (n3 PUFA)**	0.02 ± 0.00	ND
**SFA**		92.89 ± 0.06	87.91 ± 0.08	**SFA**		29.31 ± 0.09 *	54.68 ± 4.231 *
**UFA**		7.11 ± 0.06	12.09 ± 0.08	**UFA**		70.69 ± 0.09	45.32 ± 4.231
**MUFA**		5.62 ± 0.10	9.78 ± 0.05	**MUFA**		31.94 ± 0.05	20.70 ± 0.520
**PUFA**		1.49 ± 0.05	2.32 ± 0.03	**PUFA**		38.75 ± 0.05	24.62 ± 4.111
**n3PUFA**		0.87 ± 0.06	1.28 ± 0.04	**n3PUFA**		28.91 ± 0.07	16.78 ± 4.692
**n6PUFA**		0.62 ± 0.05	1.04 ± 0.02	**n6PUFA**		9.83 ± 0.05	7.84 ± 0.606
**n6/n3**		0.71 ± 0.09	0.82 ± 0.04	**n6/n3**		0.34 ± 0.00	0.51 ± 0.209

Content of fatty acids defined as ND were those detected with a contribution of less than 0.005% to the total fatty acid content, while * indicates a statistically significant difference (*p* < 0.05) between SFA and UFA. (FFA = free fatty acids; PL = polar lipids; TAC = total amphiphilic content).

**Table 8:** Representative molecular species of the main classes of the polar lipid bioactives detected in the TAC extracts of kiwi juice and by-products by LC-MS analysis.

TAC Extracts from Kiwi Juice					TAC Extracts from Kiwi By-Products			
Mainclasses of PL	Elutiontime (min)	Mr	Representative Molecular Species	Proposed Structures	Elutiontime (min)	Mr	Representative Molecular Species	Proposed Structures
**DGs**	12–12.7	679.435	DG 38:2;O2	[i.e., DG 20:1/18:1]	2.629	637.3073	DG 36:1;O	[i.e., DG 18:0/18:1 or DG 16:0/20:1]
	12–12.7	585.3298	DG 34:5	[i.e., DG 16:1/18:4]	2.629	637.3073	DG O-36:2;O2	[i.e., DG 18:1/18:1 or DG 16:1/20:1]
	12–12.7	585.3298	DG O-32:0;O2	[i.e., DG 16:0/16:0]	10.241–10.838	565.3522	DG 32:1	[i.e., DG 16:0/16:1]
	17.1–17.2	635.4519	DG 36:2;O	[i.e., DG 18:1/18:1 or 18:2/18:0 ]	10.241–10.838	565.3522	DG O-32:2;O	[i.e., DG 16:1/16:1]
	17.1–17.2	635.4519	DG O-36:3;O2	[i.e., DG 18:1/18:2 or 18:3/18:0 ]	10.241–10.838	633.3401	DG 36:3;O	[i.e., DG 18:2/18:1]
	17.1–17.2	635.4519	DG O-38:1	[i.e., DG 20:1/18:0]	10.241–10.838	633.3401	DG O-36:4;O2	[i.e., DG 18:3/18:1 or DG 18:2/18:2]
					12.297	589.5187	DG 34:3	[i.e., DG 18:2/16:1 or DG 18:3/16:0]
					12.297	589.5187	DG O-34:4;O	[i.e., DG 18:4/16:0 or DG 18:3/16:1]
**PCs**	5.6–7.6	792.8564	PC 38:5	[i.e., PC 20:1/18:4]	15.199–15.829	770.8649	PC 36:2	[i.e., PC 16:1/20:1 or PC 18:1/18:1]
	8.7–9.2	792.8560	PC 38:5	[i.e., PC 20:1/18:4]	15.199–15.829	770.8649	PC O-36:3;O	[i.e., PC 18:2/18:1]
	14.1–14.6	792.8568	PC 38:5	[i.e., PC 20:1/18:4]				
	15–15.1	792.8593	PC 38:5	[i.e., PC 20:1/18:4]				
	16.2–16.5	792.8642	PC 38:5	[i.e., PC 20:1/18:4]				
**PEs**	10.2–11.7	698.4174	PE O-34:3	[i.e., PE 18:3/16:0 or PE 18:2/16:1]	15.199–15.829	770.8649	PE 38:2	[i.e., PE 18:1/20:1]
	10.2–11.7	744.4209	PE 36:1	[i.e., PE 18:1/18:0]	7.604–7.72	770.8649	PE O-38:3;O	[i.e., PE 18:2/20:1]
	10.2–11.7	744.4209	PE O-36:2;O	[i.e., PE 18:2/18:0 or PE 18:1/18:1 or PE 20:1/16:0]				

Abbreviations: PL = polar lipids; PC = phosphatidylcholine; PE = phosphatidylethanolamine; DG = Di(acyl|alkyl)glycerols.

## Data Availability

All data are contained within the article. Any further information concerning raw data (i.e., FT-IR spectra, LC–MS chromatograms and spectra) can be provided by the authors upon request.
